# Wharton’s jelly mesenchymal stem cell-based or umbilical vein endothelial cell-based serum-free coculture with cytokines supports the ex vivo expansion/maintenance of cord blood hematopoietic stem/progenitor cells

**DOI:** 10.1186/s13287-019-1502-8

**Published:** 2019-12-05

**Authors:** Qiuyang Li, Dewan Zhao, Qiang Chen, Maowen Luo, Jingcao Huang, Cao Yang, Fangfang Wang, Wenxian Li, Ting Liu

**Affiliations:** 1 Department of Hematology, Hematology Research Laboratory, West China Hospital of Sichuan University, #37 Guo Xue Xiang Street, Chengdu, 610041 Sichuan People’s Republic of China; 2 Sichuan Cord Blood Stem Cell Bank, Chengdu, Sichuan People’s Republic of China

**Keywords:** Hematopoietic stem/progenitor cells, Expansion in vitro, Serum-free culture, Cytokines, Wharton’s jelly mesenchymal stem cells, Umbilical vein endothelial cells, Long-term culture initiating cell

## Abstract

**Background:**

The umbilical cord blood (UCB) has been widely accepted as an alternative source of hematopoietic stem/progenitor cells (HSPCs) for transplantation, and its use in adults is still restricted because of low absolute numbers. To overcome this obstacle, expansion of UCB-HSPCs under feeder cell-based coculture is a promising possibility. In this study, we explored UCB-CD34+ cells ex vivo expansion using Wharton’s jelly mesenchymal stem cells (WJ-MSCs) or umbilical vein endothelial cells (UVECs) as feeder layer-based serum-free coculture system with a cocktail of cytokines.

**Methods:**

UCB-CD34+ cells were cultured in five different coculture conditions composed of umbilical cord stromal cells (WJ-MSCs or UVECs) with or without a cocktail of cytokines (SCF, FLT3L, and TPO). The cultured cells were harvested at day 10 and analyzed for phenotypes and functionalities, including total nuclear cells (TNCs), CD34+ cells, CD34+CD38− cells, colony-forming unit (CFU) for committed progenitors, and long-term culture initiating cells (LTC-ICs) for HSPCs.

**Results:**

Our work showed the numbers of TNC cells, CD34+ cells, and CD34+CD38− cells were expanded under five coculture conditions, and the feeder layer-based cocultures further promoted the expansion. The numbers of colonies of CFU-GM, CFU-E/BFU-E, and CFU-GEMM in the cocultures with cytokines were significantly higher than their counterparts at day 0 (*p* < 0.05), while no significant difference (*p* > 0.05) in those without the addition of cytokines. The numbers of LTC-ICs were increased both under the WJ-MSCs and UVECs with cytokine cocultures, but only in the UVECs group showed a significant difference (*p* < 0.05), and were decreased under conditions without cytokine (*p* < 0.05).

**Conclusion:**

Our data demonstrate that both WJ-MSCs and UVECs as feeder layer could efficiently support the expansion of UCB-CD34+ cells in synergy with SCF, FLT3L, and TPO under serum-free culture condition. The UVECs combined with the 3GF cytokine cocktail could maintain the growth of LTC-ICs derived from UCB-CD34+ cells and even expand to some extent.

## Introduction

Since the first umbilical cord blood (UCB) transplantation was successful in 1988 [1], UCB has been as an alternative source of hematopoietic stem/progenitor cells (HSPCs) for transplantation of malignant and nonmalignant hematologic diseases. However, the amount of HSPCs in a single unit cord blood is insufficient for transplant in the most of adult patients, and the application of cord blood HSPCs remains a major limitation [2]. Therefore, different strategies have been employed to increase the number of UCB-HSPCs while maintaining their repopulating capacity. HSPCs maintain their stemness by interacting with stromal cells and extracellular matrix through cell-to-cell contact and paracrine factor secretion [3]. The microenvironment of the umbilical cord, the UCB-HSPCs residing in, is different from that in the bone marrow, which is not completely similar to bone marrow stromal cells, instead by other stromal cells, mainly including Wharton’s jelly mesenchymal stem cells (WJ-MSCs) and umbilical vein endothelial cells (UVECs). WJ-MSCs not only express cell markers of BM-MSC, but also additionally express many molecules involved in HSPC expansion and interaction, such as G-CSF, GM-CSF, and CD117 [4]. UVECs and HSPCs can express some common surface markers and transcription molecules, such as CD34, endomusin, VE-cadherin, CD31 (PECAM), Runx1, and GATA-2, to support the proliferation and differentiation of CD34+ cells in vitro [5]. These advantages make WJ-MSCs or UVECs as a preferable feeder layer choice for UCB-HSPC expansion in vitro. Regarding the concern of culture media containing animal-derived components, expansion of human HSPCs under serum-free conditions using stromal layers of human origin would have significant clinical applications [6]. In this study, we compared the effects between WJ-MSCs and UVECs cocultured with UCB-CD34+ cells under the serum-free conditions with three growth factors cocktail (stem cell factor, SCF; Flt3-ligand, FLT3L; and thrombopoietin, TPO), to explore the effects on stemness maintenance and ex vivo expansion of UCB-HSPCs.

## Method

### Isolation and characterization of WJ-MSCs and UVECs from the umbilical cord

Umbilical cord samples were collected from healthy full-term deliveries after obtaining informed consent from them as donation for research. The procedures of isolation and characterization of WJ-MSCs were performed as we earlier described in Zhao’s article [7]. By FACScan identification, WJ-MSCs expressed CD90, CD105, and CD166 and were negative for CD45, CD34, and HLA-DR. UVECs were isolated according to the following procedure: the umbilical cord sample was rinsed with normal saline to remove residual blood. It was then cut off along the umbilical vein. The vein wall was digested by collagenase, and the reaction was terminated with FBS. The primary cells were cultured in a 10.0-cm dish, using endothelial growth medium (EGM-2 Bulletkit, Lonza), incubated at 37 °C and 5% CO_2_ until the primary cells reached confluence. Isolated UVECs were seeded in 25-cm^2^ tissue culture flasks or culture dishes and cultivated in EBM medium. The primary cells were detached with trypsin-EDTA (0.25%) (HyClone Laboratories Logan, UT, USA), and the reaction was terminated with FBS to passage. The phenotypic characterization on the second to fourth passage UVECs was assayed for CD31-FITC, CD309-PCY7, CD34-PCY7, CD45FITC (eBioscience, San Diego, USA), and VWF-FITC (AbD Serotec, Kidlington, UK) via FACScan flow cytometer (Beckman Coulter, USA) according to the manufacturer’s instructions. The UVECs expressed CD31, CD309, and VWF and were negative for CD45 and CD34.

### Isolation and purification of CD34+ cells from the umbilical cord blood

The umbilical cord blood was collected from normal full-term delivery after obtaining informed consent from the mothers as a donation for banking, and only cord blood samples not appropriate for banking (< 100 ml) were used in our experiments. The procedures of isolation and characterization of CD34+ cells were performed as we earlier described in Zhao’s article [7]. The phenotypic characterization on CD34+ cells was assayed using the flow cytometer for CD34-PCY7, CD45-FITC, and CD38-APC (eBioscience, San Diego, USA). The percentages of CD34+ cells and CD34+CD38− cells were 0.952 ± 0.025 and 0.105 ± 0.070 for each cord blood unit (mean with SD, *n* = 4).

### Establishment of cocultures of CD34+ cells with WJ-MSCs and UVECs in serum-free medium

CD34+ enriched cells from the cord blood were seeded in five coculture conditions for 10 days incubation with StemSpan™ SFEM serum-free medium (Stemcell Technologies, Vancouver, Canada). WJ-MSCs or UVECs at passages 2 to 4 were plated into 12-well plates (8 × 10^4^/well) for 24 h before seeding CD34+ cells. WJ-MSCs were radiated (25 Gy) to prevent from overgrowth, and UVECs were not given radiation, since they had poor growth status after radiation. A cocktail of three growth factors was added in the medium consisting of 100 ng/ml each of FLT3L, SCF, and TPO (PeproTech, USA) according to de Lima’s report [8]. Different coculture conditions of the five groups were as follows:

1. UMC: 5000 UCB-CD34+ cells were seeded in 12-well plates in 1-ml medium under the WJ-MSCs and 3GF cytokine coculture.
2. UEC: 5000 UCB-CD34+ cells were seeded in 12-well plates in 1-ml medium under the UVECs and 3GF cytokine coculture.
3. UC: 5000 UCB-CD34+ cells were seeded in 12-well plates in 1-ml medium under the 3GF cytokine coculture, without a cell feeder.
4. UM: 40000 UCB-CD34+ cells were seeded in 12-well plates in 1-ml medium under the WJ-MSC coculture, without cytokines.
5. UE: 40000 UCB-CD34+ cells were seeded in 12-well plates in 1-ml medium under the UVEC coculture, without cytokines.

U means UCB-CD34+ cells; M, Wharton’s jelly mesenchymal stem cells; E, umbilical vein endothelial cells; and C, cytokine cocktail combined with 100 ng/ml each of SCF, FLT3L, and TPO.

Since proliferation of CD34+ cells in the coculture conditions without cytokines was much slower than that in the culturing conditions with cytokines, we seeded more CD34+ cells in the last two groups. Köhler used the similar strategy in their research to overcome the different expansion rate [9]. One-milliliter medium with cytokines was added to UMC, UEC, and UC groups, as control, and 1-ml medium without cytokines was added to UM and UE groups. Cells were incubated at 37 °C and 5% CO_2_ for 10 days. The medium was replaced on day 7 for all groups. After 10 days, both the nonadherent and adherent fraction cells were collected and analyzed for various parameters such as phenotypes and functionalities.

### Flow cytometry analysis

At day 10, we harvested the nonadherent and adherent cells from five different culture conditions, and the total nucleated cells (TNCs) counted via trypan blue staining method. Flow cytometry staining was performed with CD34-PECY7, CD45-FITC, and CD38-APC (eBioscience, San Diego, USA) antibodies, then samples were analyzed using a FACScan flow cytometer (Beckman Coulter, USA). For each sample, at least 10,000 events were recorded. The isotype antibodies were used to determine the level of nonspecific binding.

### Colony-forming cell (CFC) assay

We harvested cells from each group at day 10, and cells were seeded in semisolid culture (H4434, Stem Cell Technologies, Vancouver, Canada) following the manufacturer’s instructions for the colony-forming unit (CFU) assay. After incubation at 37 °C under 5% CO_2_ at 100% humidity for 14 days, the burst-forming unit-erythroid (BFU-E), colony-forming unit-erythroid (CFU-E), colony-forming unit-granulocyte/macrophage (CFU-GM), and colony-forming unit-granulocyte, erythroid, macrophage, megakaryocyte (CFU-GEMM) levels were scored under an inverted microscope. The numbers of CFU per 5000 UCB-CD34+ cells before cultivation (day 0) and after harvest (day 10) were calculated. Each colony-forming unit is equivalent to a colony-forming cell (CFC).

### Long-term culture initiating cell (LTC-IC) assay

M2-10B4, a murine fibroblast cell line, was used as a feeder layer. At least 24 h before assay, M2-10B4 cells were radiated (80 Gy) and seeded in six-well plates as feeder cells (2.5 × 10^5^/well). The plates were coated with collagen solution (StemCell Technologies, Vancouver, Canada). The cells harvested from different coculture conditions at day 10 were resuspended with H5100 containing 10^−6^ M hydrocortisone (StemCell Technologies, Vancouver, Canada) and then seeded into the plate with the feeder layers. Half of the medium was replaced in weekly intervals. Both nonadherent and adherent cells were harvested at week 5 and reseeded in semisolid culture (H4434, StemCell Technologies, Vancouver, Canada) for CFC assay. After 16 days, colonies were scored under an inverted microscope. The LTC-IC number was calculated according to the manufacturer’s instructions. Each eight CFC colonies correspond to one LTC-IC, and the LTC-IC numbers per 5000 UCB-CD34+ cells before cultivation (day 0) and after harvesting (day 10) were calculated.

### Statistical analysis

The statistical differences between each group were analyzed using the GraphPad 7.0 statistical software for all the experiment data. The comparison was analyzed between two groups with an independent sample *t* test. The values were plotted as mean ± standard deviation. Probability values *p ≤ 0.05 was considered statistically significant.

## Results

### The influence of cytokines and feeder cells (WJ-MSCs or UVECs) on cocultures of UCB-HSPCs

**Fig. 1:**
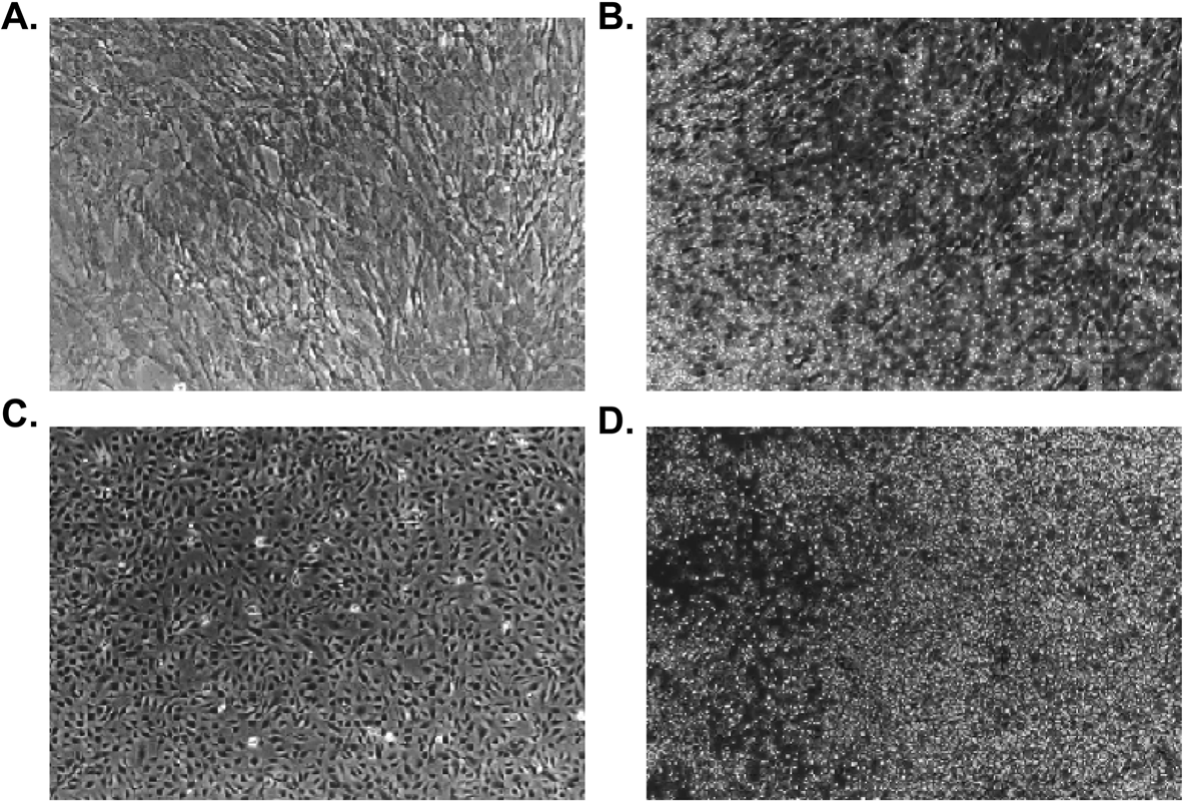
The proliferation status of UCB-HSPCs under a microscope. **a** UCB-CD34+ cells with WJ-MSC coculture in the presence of cytokines at day 0. **b** UCB-CD34+ cells with WJ-MSC coculture in the presence of cytokines at day 10. **c** UCB-CD34+ cells with UVEC coculture in the presence of cytokines at day 0. **d** UCB-CD34+ cells with UVEC coculture in the presence of cytokines at day 10

**Fig. 2:**
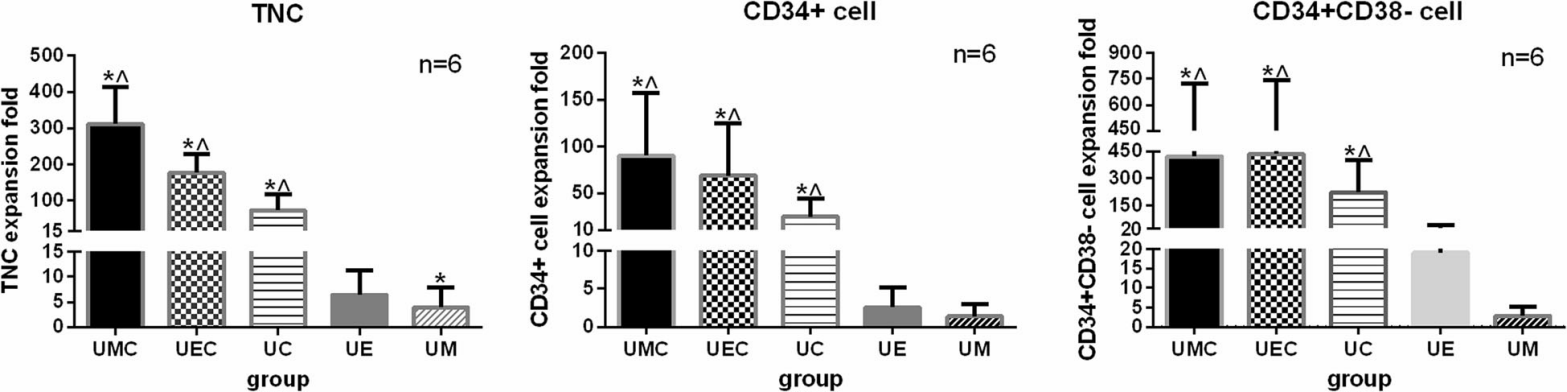
The expansion folds of TNCs, CD34+ cells, and CD34+CD38− cells under different coculture conditions. The asterisk indicates *p* < 0.05, comparing the counts before cultivation (day 0) and after harvest (day 10), and the caret indicates *p* < 0.05, comparing the counts with cytokine groups (UMC, UEC, UC) and without cytokine groups (UM, UE) at day 10

To explore the influence of cytokines with feeder cells on the proliferation of UCB-HSPCs, we performed 10-day cocultures under different conditions described above (UMC, UEC, UC, UE, UM) and observed the growth of UCB-HSPCs under a microscope (Fig. 1.). The total numbers of TNCs, CD34+ cells, and CD34+CD38− cells were evaluated at day 10. Based on the cell counts and percentage, we calculated the numbers of different cell subtype proliferation and their expand folds. The results show that “3GF cocktail” (100 ng/ml each of FLT3L, SCF, and TPO) could significantly simulate the proliferation of UCB-HSPCs. The numbers of TNCs, CD34+ cells, and CD34+CD38− cells largely increased on the coculture conditions with cytokines (UMC, UEC, UC) and could maintain growth with feeder cells (UE and UM) without adding cytokines. Comparing the counts before cultivation (day 0) with those after harvest (day 10), and with cytokines (UMC, UEC, UC) or without cytokines (UM, UE), the folds of expansion of TNCs, CD34+ cells, and CD34+CD38− cells are summarized in Table 1 and Fig. 2. The results suggested that the umbilical cord stromal cells could efficiently support expansion of stem and progenitor cells in synergy with SCF, FLT3L, and TPO under serum-free culture condition.

**Table 1:** The expansion folds of UCB-CD34+ cells under different coculture conditions

***N*** = 6	UMC	UEC	UC	UM	UE
TNCs	312.52 ± 100.68*^,^^	177.57 ± 51.16*^,^^	74.00 ± 42.94*^,^^	4.03 ± 3.82	6.42 ± 4.87*
CD34+ cells	90.17 ± 67.00*^,^^	68.72 ± 56.310*^,^^	24.50 ± 19.49*^,^^	1.40 ± 1.65	2.60 ± 2.54
CD34 + CD38- cells	423.07 ± 300.43*^,^^	435.15 ± 308.16*^,^^	222.99 ± 179.30*^,^^	2.92 ± 2.39	19.10 ± 22.88

*Comparing the cell counts before cultivation (day 0) and after harvest (day 10), the folds of expansion of TNCs, CD34+ cells, and CD34 + CD38− cells, *p* < 0.05

^^^Comparing the cell counts with cytokines (UMC, UEC, UC) and without cytokines (UM, UE) at day 10, the folds of expansion of TNCs, CD34+ cells, and CD34+CD38− cells, *p* < 0.05

### The proliferation of committed progenitors in different coculture conditions

**Fig. 3:**
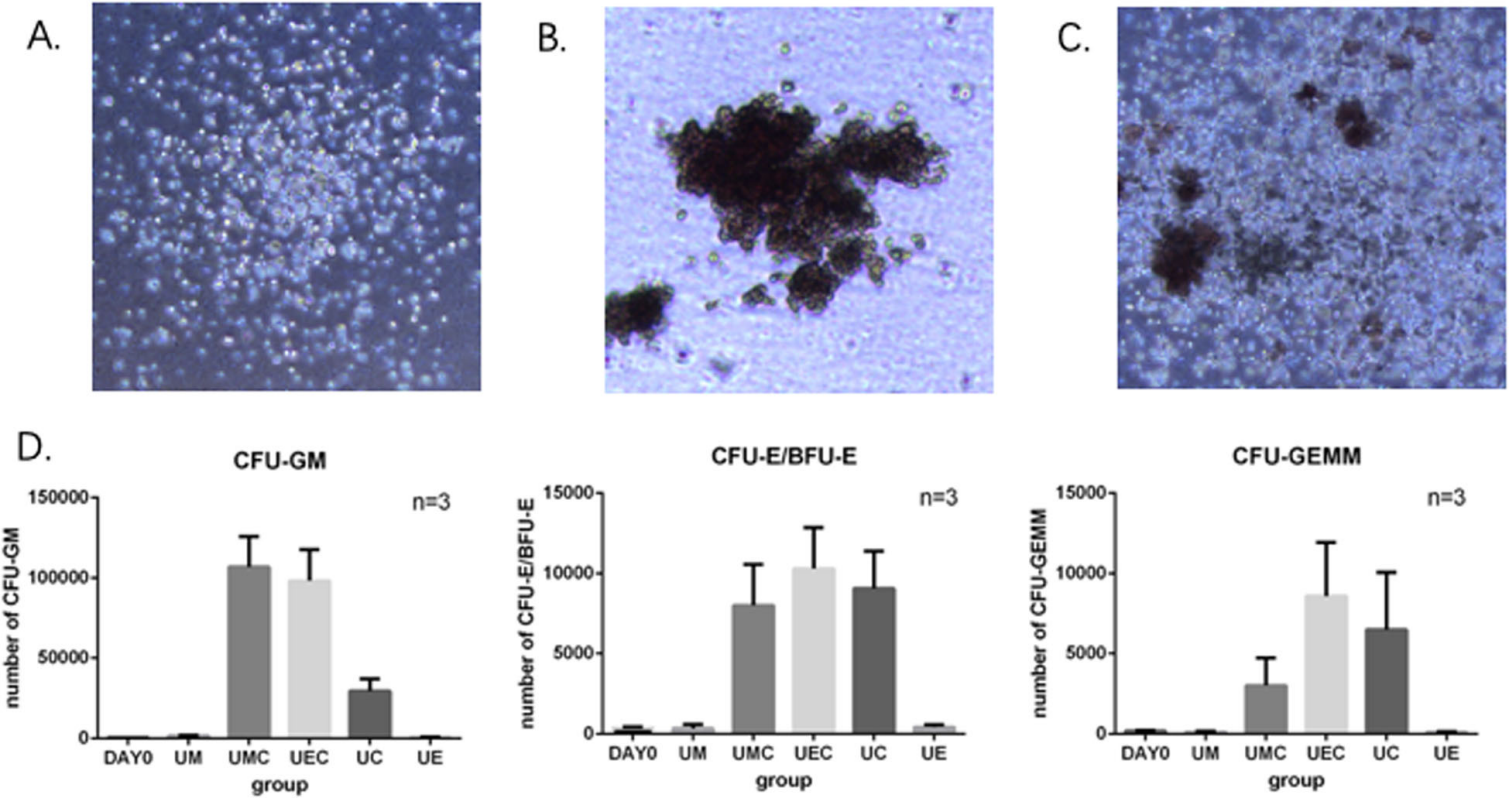
The proliferation of committed progenitors in different coculture conditions. Morphology of CFUs: **a** CFU-GM, colony-forming unit-granulocyte/macrophage; **b** CFU-E/BFU-E, colony-forming unit-erythroid/burst-forming unit-erythroid; **c** CFU-GEMM, colony-forming unit-granulocyte/erythroid/macrophage/megakaryocyte; and **d** the expansion numbers of CFU-GM, CFU-E/BFU-E, and CFU-GEMM in different coculture conditions, which were significantly higher in adding cytokine groups (UMC, UEC, and UC) than in their counterparts at day 0 (*p* < 0.05), while there was no significant difference (*p* > 0.05) in groups without cytokine (UM, UE)

We evaluated the numbers of committed progenitors in different coculture conditions through colony-forming assays (CFC assays) and calculated the numbers of colonies derived from 5000 initial CD34+ cells. The numbers of colonies of CFU-GM, CFU-E/BFU-E, and CFU-GEMM under coculture conditions containing cytokines (UMC, UEC, and UC) were significantly higher than their counterparts at day 0 (*p* < 0.05), while there was no significant difference (*p* > 0.05) under coculture conditions without cytokines (UM, UE). These results demonstrate that the “3GF cocktail” could promote the proliferation of different kinds of committed progenitors (Fig. 3.).

### The long-term reconstruction capacity maintenance

**Fig. 4:**
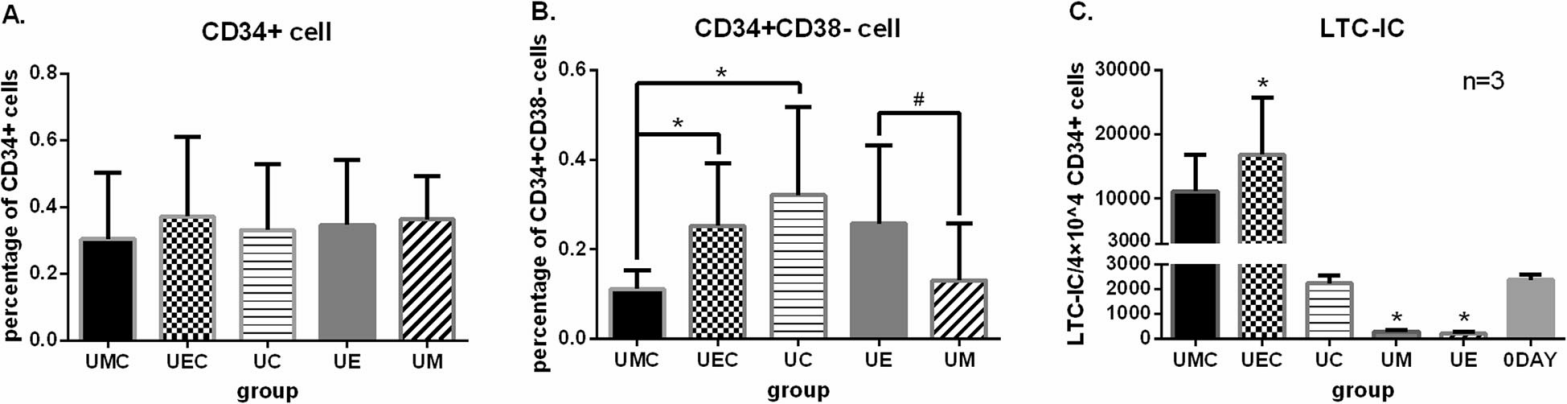
The long-term reconstruction capacity maintenance. **a** The percentage of CD34+ cells under different coculture conditions, and no significant difference was observed (*p* > 0.05). **b** The percentage of CD34+CD38− cells under different coculture conditions. The asterisk indicates *p* < 0.05, comparing the cells under the UEC and UC conditions with those under the UMC condition. The number sign indicates *p* < 0.05, comparing the cells under the UE with those under the UM conditions. **c** The numbers of LTC-ICs in different coculture conditions. The asterisk indicates *p* < 0.05, comparing the numbers of LTC-ICs at day 10 with those at day 0, that showed the results of the increase in UEC and decrease in UM and UE conditions

Phenotypically, UCB-HSPCs are identified by the expression of CD34 surface glycophosphoprotein and by the absence of all lineage-specific markers [10]. This primitive population represents about 0.1~0.4% in TNCs of UCB. Within the Lin−/CD34+ cell population, the CD38 marker is used to differentiate multipotent progenitors (CD38−) and committed progenitors (CD38+) [11]. The subpopulation CD34+/CD38− represents about 10% of UCB-HSPCs. In our study, comparing the harvested cells at day 10 among different coculture groups (UMC, UEC, UC, UE, UM) via flow cytometry analysis, the percentages of CD34+ cells were 0.30 ± 0.20, 0.37 ± 0.24, 0.33 ± 0.20, 0.35 ± 0.20, and 0.36 ± 0.13, and there was no statistical significance (*p* > 0.05) (Fig. 4a), and the percentages of CD34+CD38− cells were 0.11 ± 0.04, 0.25 ± 0.14, 0.32 ± 0.20, 0.26 ± 0.17, and 0.13 ± 0.13 respectively. A higher percentage of CD34+CD38− cells was shown in UEC than in UMC coculture (*p* < 0.05); similarly, the percentages of CD34+CD38− cells in UE condition were higher than those in UM (*p* < 0.05) (Fig. 4b). The LTC-IC assay was performed to evaluate the long-term reconstruction capacity maintenance of UCB-HSPCs in vitro, and it partially reveals the function of hematopoietic stem cells. We performed LTC-IC assays at day 0 for the CD34+ cells isolated from the cord blood and at day 10 for the cells harvested from different coculture conditions (UMC, UEC, UC, UM, UE). Comparing the numbers of LTC-ICs derived from day 10 with day 0 assessment, the results showed that the numbers of LTC-ICs increased both in the UMC and UEC coculture conditions, but only significant difference in the UEC group (*p* < 0.05), and decreased in UM and UE conditions (*p* < 0.05). In addition, the number of LTC-ICs seemed not to change after coculture in UC condition. These indicate that the “3GF cocktail” might play more role on the maintenance of LTC-ICs in our coculture system (Fig. 4c).

## Discussion

UCB-HSPC is considered an alternative source of HSPCs for hematopoietic stem cell transplantation (HSCT), because of the easy availability, tolerance of HLA mismatch, and low incidence of graft versus host disease (GVHD). Moreover, UCB-HSPCs have a higher frequency of progenitors with greater clonogenic potential compared to adult counterpart from BM and PB. Unfortunately, the yield of UCB-HSPCs from a single unit is insufficient for the transplant in most of adult patients. Therefore, several attempts have been made to expand UCB-HSPCs in vitro by using combinations of specific media, cytokines, growth factors, and recently also with hematopoietic stromal cells as feeder layer coculture. Some results had showed that using a cocktail of cytokines combined with other methods could promote the expansion of UCB-HSPCs. Delaney et al. used cytokine cocktail containing high dose of SCF, FLT3L, TPO, IL-3, and IL-6 combined with Delta1ext-IgG and got 6.2-fold increase of SCID repopulating cells (SRCs) [12]. Ueda et al. got 4.2-fold increase in UCB-CD34+ cell expansion via using a cytokine recipe of SCF, FLT3L, TPO, IL-3, IL-6, and s-IL-6R with 20% FBS [13]. Butler et al. used E4ORF-transducted UVECs combined with SCF, IL-3, and TPO which caused 7.7-fold increase in SRCs [14]. SCF, FLT3L, and TPO are called early-acting cytokines, and they act preferentially on more primitive progenitors of hematopoietic cells [15]. FLT3 is mainly expressed on the surface of HSPCs, and FLT3L is secreted by and expressed on the surface of hematopoietic stromal cells. Receptor tyrosine kinase (RTK) pathway can be activated to promote expansion of HSPCs in vitro via FLT3 interacting with FLT3L [16]. SCF can stimulate expansion of HSPCs by activating c-kit receptor on the cells’ surface under the presence of FLT3L and TPO coculture conditions [17], and TPO may act to UCB-HSPCs by upregulating HoxB4 gene expression, which promote HoxA9 transport from cytoplasm to nucleus, and upregulate the expression of vascular endothelial growth factor [18]. Zaker et al. used each of 100 ng/ml SCF, FLT3L, and TPO to expand CD34+ cells derived from the cord blood with BM-MSCs and a high affinity copper (Cu) chelator TEPA, and their results showed that the CD34+CD38− cells were expanded [19]. For this reason, the “3GF cocktail” had been used for UCB-HSPC ex vivo expansion without the addition of serum or with other late acting cytokines. Our results showed that adding “3GF cocktail” in coculture systems could stimulate expansions of TNCs, CD34+ cells, CD34+CD38− cells, and CFCs of different lineage. However, combination of “3GF cocktail” alone could not lead to expansion of LTC-ICs. It might be related to the effect of cytokines recruiting pluripotent, dormant progenitors into cell cycle. It has been reported that cytokines may promote the expansion of HSPCs in vitro by reducing expression of cell cycle-dependent kinase inhibitors (CDKIs) and promoting the cells from G1 to S phase [20, 21]. This effect was associated with inevitable loss of stem cell function.

Since most of the studies have confirmed that the cytokine-driven expansion conditions are accompanied by concomitant cell differentiation, using hematopoietic stromal cells as feeder cells, coculture has been a more natural approach to augment the number of UCB-HSPCs [22]. In human hematopoietic microenvironment, stromal cells could support hematopoiesis by two mechanisms: direct cell-to-cell contact and secretion of specific factors. Mishima et al. demonstrated that cell-to-cell contact was crucial to promote expansion of stem cell progenitors [23]. This cell-supportive interaction could be mimicked by an in vitro model where HSPCs can cocultured in [24]. Unlike cytokines, stromal cells maintain the growth of HSPCs by reducing apoptosis of HSPCs and maintaining cells in G0/G1 phase and further increase cell number in the existence of additional cytokines [25]. Butler et al. used the E4ORF-transducted HUVECs as feeder cells to combine with cytokines, which could cause expansion of SRCs, and showed higher expansion folds and greater engraftment potential compared with the cells cultured alone in the action of cytokines [14]. Magin et al. compared the feeder potential of three primary cell types: BM-MSCs, WJ-MSCs, and UVECs. They found that all three cells had a comparable potential to support UCB-CD34+ cell expansion, with WJ-MSCs even superior to the other two cell types [26]. In our study, UCB-CD34+ cells and cord-derived stromal cells were cocultured in serum-free medium. It revealed either WJ-MSCs or UVECs as coculture feeder layer without additional cytokines could maintain the growth of UCB-HSPCs. In the existence of “3GF cocktail,” WJ-MSCs and UVECs could further promote the expansion of UCB-HSPCs in vitro. Comparing the counts of before cultivation (day 0) with after harvest (day 10) in various coculture conditions (UMC, UEC, UC, UM, UE), the folds of expansion of CD34+CD38− cells under UMC and UEC conditions were more than UC, UM, and UE conditions. The similar effects were also observed on other stromal cells derived from the bone marrow, cord tissue, and placenta in other researches [27–30].

As a main hematopoietic microenvironment of the cord blood, WJ-MSCs and UVECs might take part in the sustainability of the stem cell function of UCB-HSPCs in vivo. Bakhshi et al. reported that WJ-MSCs, similarly to BM-MSCs, effectively maintained UCB-CD34+ cells as demonstrated by the capability to form colonies in the LTC-IC assay [31]. In our study, the number of LTC-ICs under the UEC coculture was increased significantly compared with its counterpart at day 0, and revealed the similar tendency under the UMC condition, though without statistic difference. Compared with the outcome of the UC, UM, and UE conditions, WJ-MSCs and UVECs seem to be helpful for maintaining the stem cell function of UCB-HSPCs ex vivo in the existence of additional cytokines. Of course, the golden standard to confirm the functional hematopoietic stem cells is replantation in a NOD/SCID mouse transplant model, the LTC-ICs are means of the earliest HSPCs that could be identified in vitro [32]. Of note is that these two kinds of feeder cells also showed some difference on the phenotypes of HSPCs in our data. The higher percentages of CD34+CD38− cells were shown in UEC than in UMC coculture. Since the sample size was small, this result should be further validated by more experiments. Raynaud et al. compared the MSCs derived from the placenta (PL-MSCs) with Akt-activated HUVECs (E4ORF1, referred here as E4+ECs) as feeder layers, and the results showed a clear preference of hematopoietic cells for interacting with endothelial cells indicating either the role of membrane-bound factors or stronger chemo-attraction by endothelial cells than PL-MSCs. Transcriptomic analysis demonstrated many differences between the two feeders. The main difference in terms of angiocrine factor was the overexpression of Notch ligands by E4+ECs [5]. However, the observed functional differences are not based on a single molecule, but most likely rely on the fine-tuning of factors involved in regulating stemness, expansion, and differentiation. Future studies should focus on identifying the essential factors of the endothelial cells responsible for the maintenance and expansion of UCB-HSPCs.

## Conclusion

Our results indicate that using two human umbilical cord stromal feeder cells (WJ-MSCs and UVECs) could effectively support expansion of UCB-CD34+ cells in synergy with a cocktail of cytokines containing 100 ng/ml each of SCF, FLT3L, and TPO under serum-free culture condition in vitro, and meanwhile preserve the capability of UCB-CD34+ cells to form colonies in the LTC-IC assay. The UVECs combined with the 3GF cytokine cocktail could maintain the growth of LTC-ICs from UCB-CD34+ cells and even expand to some extent. As one of the common methods to expand hematopoietic cells in vitro, this coculture system can be used as a bionic model for hematopoietic stem cell research and, after a further improvement, might possibly be suitable for using in a potentially clinically applicable system for expansion of UCB-HSPCs.

## Data Availability

Please contact the author for data requests.

## Abbreviations

UCB: Umbilical cord blood
HSPCs: Hematopoietic stem cells/progenitor cells
WJ-MSCs: Wharton’s jelly mesenchymal stem cells
UVECs: Umbilical vein endothelial cells
BM-MSC: Bone marrow-derived mesenchymal stem cells
PL-MSCs: Placenta-derived mesenchymal stem cells
TNC: Total nuclear cell
MNC: Mononuclear cell
CFU: Colony-forming unit
BFU-E: Burst-forming unit-erythroid
CFU-GM: Colony-forming unit-granulocyte/macrophage
CFC: Colony-forming cell
LTC-IC: Long-term culture initiating cell
HSCT: Hematopoietic stem cell transplantation
SCF: Stem cell factor
TPO: Thrombopoietin
FLT3L: Flt3-ligand
RTK: Receptor tyrosine kinase
